# Cross resistance to diverse anticancer nicotinamide phosphoribosyltransferase inhibitors induced by FK866 treatment

**DOI:** 10.18632/oncotarget.24731

**Published:** 2018-03-27

**Authors:** Yoko Ogino, Akira Sato, Fumiaki Uchiumi, Sei-ichi Tanuma

**Affiliations:** ^1^ Faculty of Pharmaceutical Sciences, Tokyo University of Science, Noda, Chiba 278-8510, Japan

**Keywords:** drug resistance, FK866, NAD^+^ biosynthesis, NAMPT, point mutation

## Abstract

Cross-resistance to drugs remains an unsolved problem in cancer chemotherapy. This study elucidates a molecular mechanism of cross-resistance to diverse inhibitors of nicotinamide phosphoribosyltransferase (NAMPT) with anticancer activity. We generated a variant of the human colon cancer cell line HCT116, HCT116R^FK866^, which exhibited primary resistance to the potent NAMPT inhibitor FK866, and was approximately 1,000-fold less sensitive to the drug than the parental HCT116. HCT116R^FK866^ was found to be cross-resistant to diverse NAMPT inhibitors, including CHS-828, GNE-617, and STF-118804. Whole-exon sequencing revealed two point mutations (H191R and K342R) in NAMPT in HCT116R^FK866^, only one of which (K342R) was present in the parental HCT116. Importantly, the protein level, NAMPT enzyme activity, and intracellular NAD^+^ level were similar between HCT116R^FK866^ and HCT116. Hence, we investigated NAMPT-binding partners in both cell lines by focused proteomic analyses. The amount of NAMPT precipitated with anti-NAMPT monoclonal antibody was much higher in HCT116R^FK866^ than in the parental. Furthermore, in HCT116, but not in HCT116R^FK866^, NAMPT was revealed to interact with POTE ankyrin domain family member E and beta-actin. Thus, these results suggest that NAMPT usually interacts with the two partner proteins, and the H191R mutation may prevent the interactions, resulting in resistance to diverse NAMPT inhibitors.

## INTRODUCTION

NAD^+^ is an essential redox cofactor in energy metabolism, including glycolysis, the TCA cycle, and oxidative phosphorylation [1, 2]. In addition, it serves as a substrate for multiple enzymes, such as sirtuin and poly (ADP-ribose) polymerase, involved in cellular signaling processes [2], calcium homeostasis [3], gene regulation [4], genome integrity [5], and cell survival or death [6]. The main pathways for NAD^+^ biosynthesis include the *de novo* pathway, from tryptophan, and two salvage pathways, from nicotinamide (NAM) and nicotinic acid (NA) [2, 7]. Many cancer cells have a much higher rate of NAD^+^ turnover, and primarily use the NAM salvage pathway. Accordingly, nicotinamide phosphoribosyltransferase (NAMPT), the rate-limiting enzyme of the salvage pathway, is considered an attractive target for the development of anticancer drugs [1, 8].

The main form of human NAMPT is a 491–amino acid protein (molecular weight, ∼55 kDa) that catalyzes a condensation reaction between NAM and phosphoribosyl pyrophosphate (PRPP) to yield nicotinamide mononucleotide (NMN). NAMPT functions as a homodimer belonging to the family of type II phosphoribosyltransferases, with two identical active sites responsible for NAM and PRPP binding [9, 10]. First-line NAMPT inhibitors, including FK866 (also known as APO866 and WK175) [11, 12] and CHS-828 (also known as GMX1778) [13–16], have already entered clinical trials for anticancer chemotherapy. A number of new NAMPT inhibitors, including GNE-617 [1, 17] and STF-118804 [18], are in the preclinical stages [8].

Continuous exposure of cancer cells to NAMPT inhibitors can result in acquired resistance to these drugs, often caused by mutations in NAMPT [19–21]. For example, the G217R point mutation, first detected in inhibitor-resistant HCT116 human colon cancer cells, results in a 2,500-fold shift in the 50% effective concentration (EC_50_) of CHS-828 relative to parental HCT116 cells, with no associated change in the level of NAMPT protein [20]. NAMPT mutations that confer resistance to specific NAMPT inhibitors, such as FK866 and CHS-828, include G217R, H191R, D93del, and Q388R [21]. Based on the wild-type NAMPT structure, the side chains of the mutated residues in G217R and H191R protrude into the inhibitor-binding pocket tunnel in NAMPT, whereas the D93del and Q388R mutations are located on the dimer interface [20, 21]. Recently, Wang *et al.* reported six point mutations (D93del, S165F, S165Y, G217R, G217A, G217V) in rhabdosarcoma RD, pancreatic cancer MIAPaCa-2, and non–small cell lung cancer NCI-H460 cells that became resistant to GNE-618 [19]. Some mutated NAMPT alleles are thought to be resistant to NAMPT inhibitors due to lack of occupancy of the tunnel-shaped cavities near the NAM-binding sites [20, 21]. The S165F mutant is 1,000-fold more resistant to GNE-618, but only 10-fold and 100-fold more resistant to FK866 and GMX1778, respectively, suggesting that NAMPT mutants are differentially affected by distinct classes of NAMPT inhibitors [19]. Moreover, cell lines harboring S165Y and G217Y are preferentially resistant to GNE-618 in comparison with GMX1778 and FK866 [19]. The precise molecular mechanisms by which cancer cells become cross-resistant to NAMPT inhibitors remain to be elucidated.

To address this issue, we established an FK866-resistant HCT116 cell line (HCT116R^FK866^) and analyzed its characteristics. Importantly, HCT116R^FK866^ cells were found to be cross-resistant to diverse classes of NAMPT inhibitors, including CHS-828, GNE-617, and STF-118804. To elucidate the molecular cause of the drug resistance, we performed whole-exon sequencing to compare the *NAMPT* gene between HCT116R^FK866^ and parental HCT116 cells. The results revealed that the point mutation H191R was present in HCT116R^FK866^, but not in parental HCT116 cells. Importantly, NAMPT protein level and enzyme activity were similar in HCT116R^FK866^ and HCT116 cells.

Next, we used a focused proteomic approach, immunoprecipitation with anti-NAMPT monoclonal antibody (mAb) followed by mass spectrometry, to identify NAMPT-binding partners in HCT116R^FK866^ and HCT116 cells. The level of precipitated NAMPT was much higher in HCT116R^FK866^ than in HCT116 cells. We also identified two NAMPT-binding proteins, POTEE and beta-actin, in HCT116 but not in HCT116R^FK866^ cells. These findings suggest that the NAMPT H191R mutation in HCT116R^FK866^ cells abolishes interactions with the two binding partners, thereby decreasing the protein’s affinity for various NAMPT inhibitors.

## RESULTS AND DISCUSSION

### Establishment of FK866-resistant HCT116 cells

To elucidate the mechanisms underlying cross-resistance to diverse NAMPT inhibitors, we generated a variant of the human colon cancer cell line HCT116 that was resistant to FK866, a potent inhibitor of NAMPT *in vitro* that has efficacy against various human tumors in *in vivo* xenograft models [11, 12]. FK866, also known as APO866 and WK175, is a clinical-stage first-line candidate [11, 12]. As in previous experiments aimed at obtaining cells resistant to NAMPT inhibitors, our initial efforts focused on generating a NAMPT inhibitor–resistant HCT116 cell line with one point mutation in the NAMPT protein.

**Figure 1:**
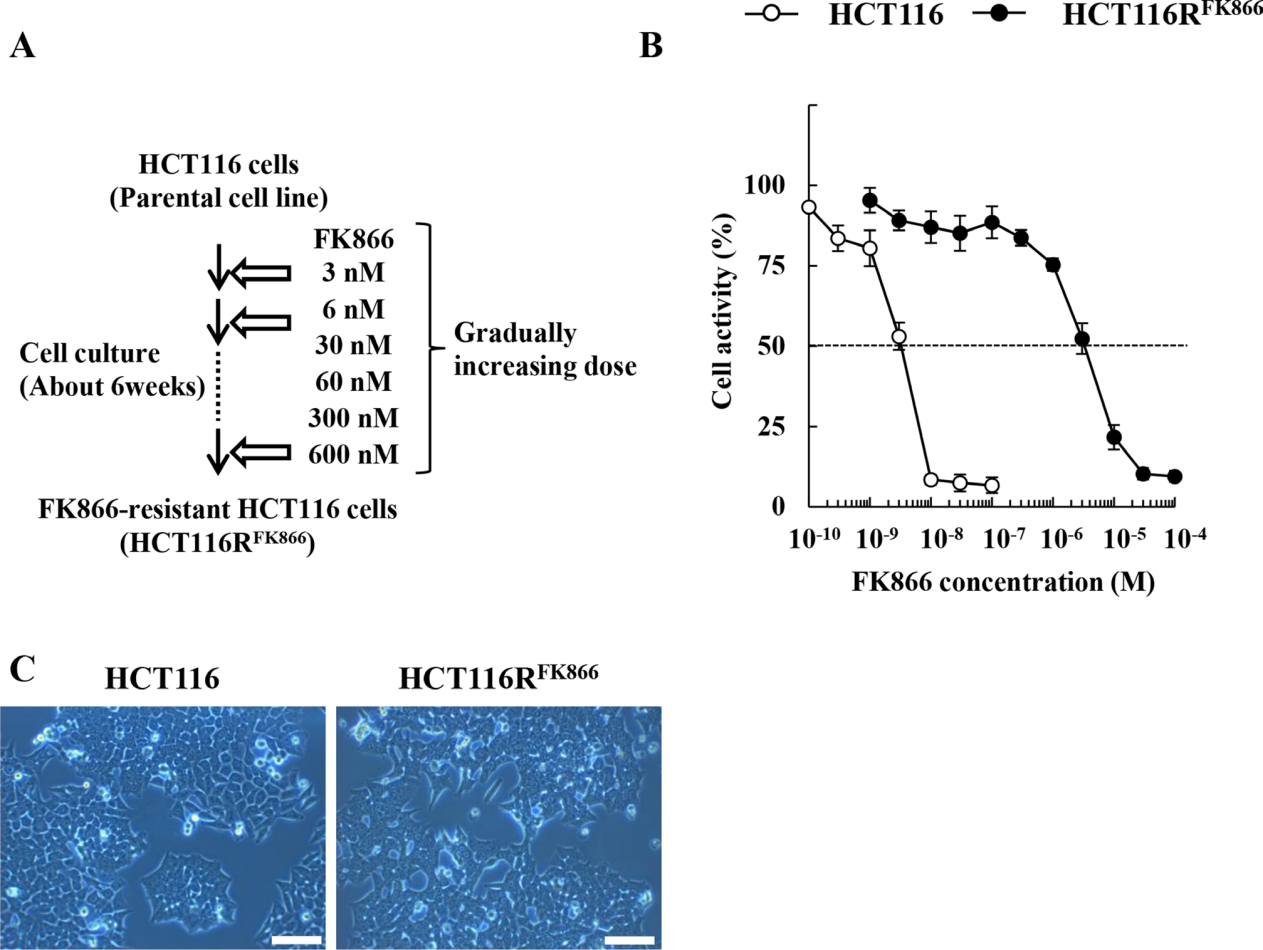
Establishment of HCT116R^FK866^, an FK866-resistant derivative of the human colon cancer cell line HCT116 (**A**) Scheme for establishment of FK866-resistant HCT116 cells (HCT116R^FK866^). (**B**) HCT116R^FK866^ and parental HCT116 cells were tested for cell viability after 72 hr treatment with FK866. Results are averages of three independent experiments with error bars showing ± SE from triplicates. (**C**) Morphological features were analyzed by Olympus CK40 microscope at 100× magnification. Scale bar = 100 µm.

By repeated exposure to stepwise increasing concentrations of FK866 over a period of about 6 weeks (Figure 1A), we established HCT116R^FK866^, an HCT116 variant resistant to the drug. The EC_50_ of FK866 in HCT116R^FK866^ cells was determined by WST-8 assay after continuous exposure for 72 hr. As shown in Figure 1B, the EC_50_ value of HCT116R^FK866^ cells was significantly higher (EC_50_ = 3,300 nM) than that of the sensitive parental HCT116 cells (EC_50_ = 3.3 nM). The resistance index (RI) was approximately 1,000 (Table 1). In addition, parental HCT116 and HCT116R^FK866^ cells have almost similar morphological features (Figure 1C).

**Table 1:** Summary of FK866 sensitivity and NAMPT mutations in HCT116R^FK866^ and parental HCT116 cells

	NAMPT mutation	EC_50_ (FK866, nM)	RI
HCT116	*mt* (K342R)	3.3	-
HCT116R^FK866^	*mt* (K342R) *mt* (H191R)	3,300	1,000 ^a^

The EC_50_ values are averages of triplicated determinations obtained from 3 independent experiments. *mt*, mutation. EC_50_, 50% effective concentration; RI, resistance index: ^a^ *P <* 0.05, vs. HCT116 cells.

### Cross-resistance to diverse NAMPT inhibitors in FK866-resistant HCT116 cells

**Figure 2:**
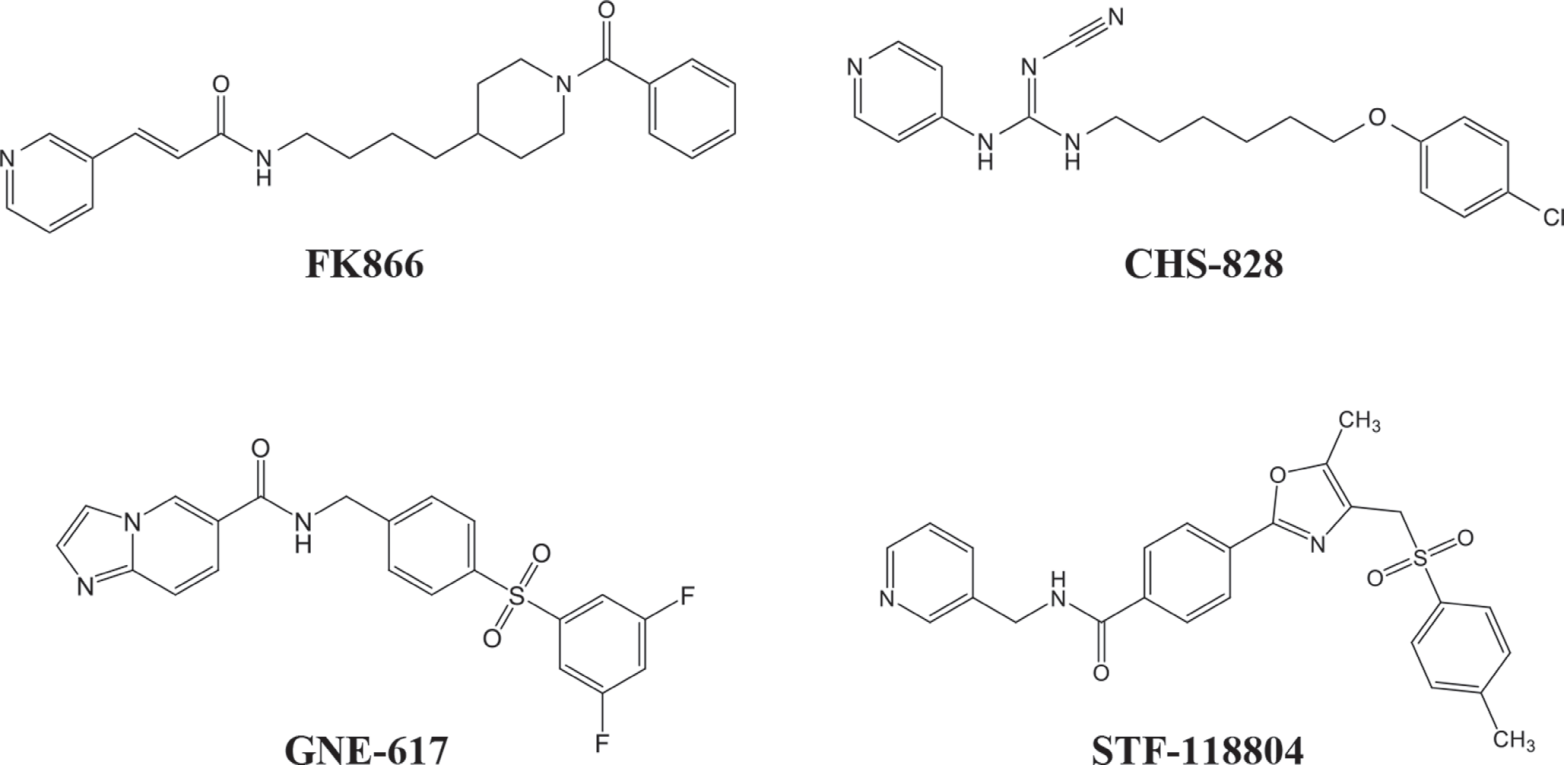
Chemical structures of specific NAMPT inhibitors Structures of (E)-N-(4-(1-benzoylpiperidin-4-yl)butyl)-3-(pyridin-3-yl)acrylamide (FK866; APO866; WK175), (E)-1-(6-(4-Chlorophenoxy)hexyl)-2-cyano-3-(pyridin-4-yl)-guanidine (CHS-828; GMX1778), N-(4-((3,5-difluorophenyl)sulfonyl)benzyl)imidazo[1,2-a]pyridine-6-carboxamide (GNE-617), and 4-[5-methyl-4-[(4-methylphenyl)sulfonylmethyl]-1,3-oxazol-2-yl]–N -(pyridin-3-ylmethyl)benzamide (STF-118804).

**Figure 3:**
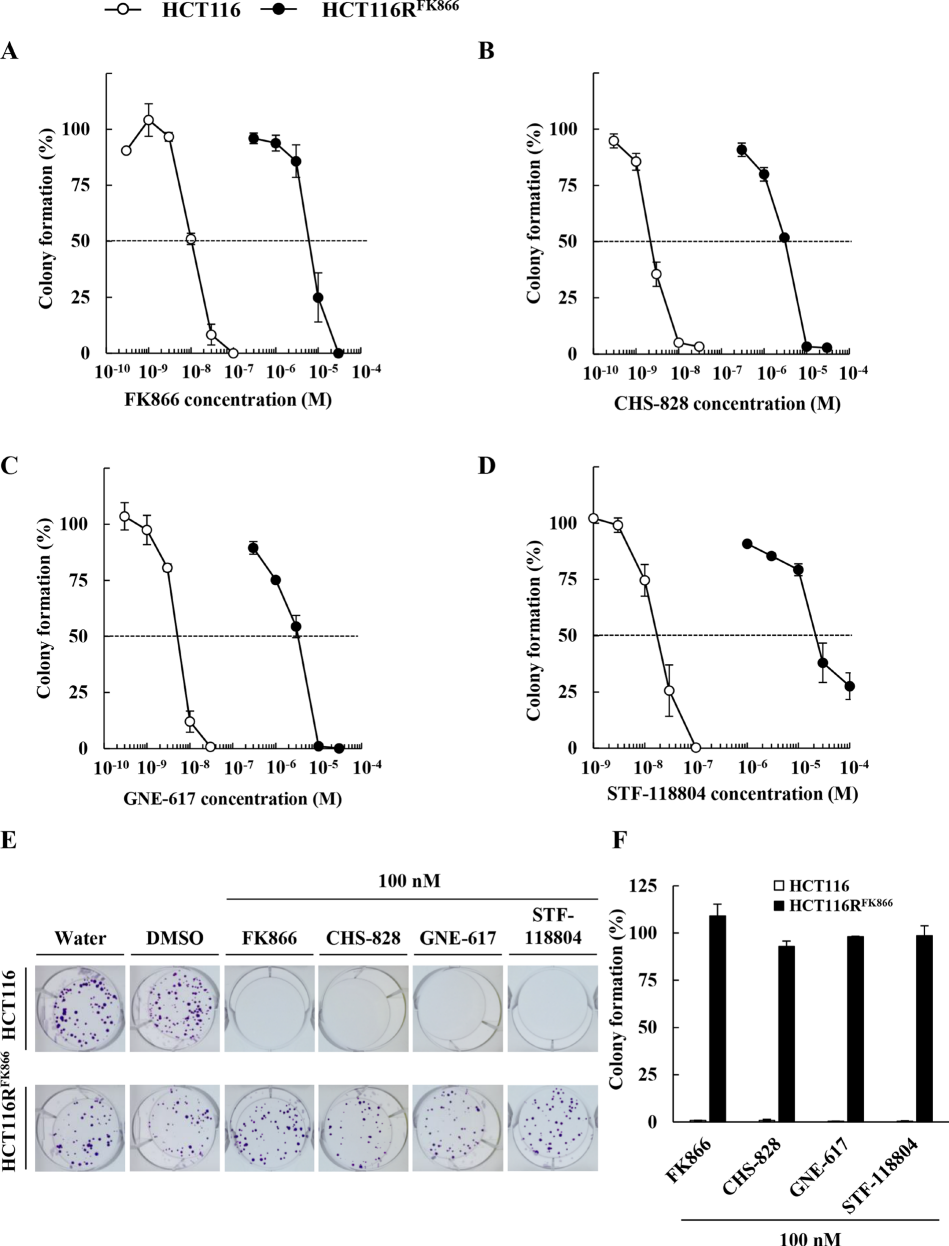
Sensitivity to diverse NAMPT inhibitors, FK866, CHS-828, GNE-617, and STF-118804, in HCT116R^FK866^ and parental HCT116 cells Colony formation by HCT116R^FK866^ and parental HCT116 cells after 10 days of treatment with (**A**) FK866, (**B**) CHS-828, (**C**) GNE-617, and (**D**) STF-118804. Results are averages of two independent experiments, with error bars showing ± SE of triplicates. (**E**) Drug sensitivities of HCT116R^FK866^ and HCT116 in the colony formation assay. HCT116R^FK866^ and HCT116 cells were treated with 100 nM each of FK866, CHS-828, GNE-617, STF-118804, and incubated for 10 days. (**F**) Colony formation (%) are averages of two independent experiments, with error bars showing ± SE of triplicates.

We next examined the effects of diverse NAMPT inhibitors, including FK866, CHS-828, GNE-617, and STF-118804, (Figure 2), on the proliferation of HCT116R^FK866^ and HCT116 cells, as determined by colony formation assays. As shown in Figure 3A, HCT116R^FK866^ cells were 627-fold (EC_50_ = 6,650 nM) more resistant to FK866 than the parental HCT116 cells (EC_50_ = 10.6 nM). Similarly, the EC_50_ values for CHS-828, GNE-617, and STF-118804 were 1,370-fold (EC_50_ = 3,150 nM), 625-fold (EC_50_ = 3,250 nM), and 1,447-fold (EC_50_ = 28,500 nM) higher, respectively, in HCT116R^FK866^ cells (CHS-828, EC_50_ = 2.3 nM; GNE-617, EC_50_ = 5.2 nM; STF-118804, EC_50_ = 19.7 nM) (Figure 3B–3F and Table 2). These results suggest that HCT116R^FK866^ cells confer cross-resistance to diverse NAMPT inhibitors, including CHS-828, GNE-617, and STF-118804.

**Table 2:** Cross-resistance of HCT116R^FK866^ cells to diverse NAMPT inhibitors, FK866, CHS-828, GNE-617, and STF-118804

	HCT116	HCT116R^FK866^	RI^a^
	EC_50_ (nM)	EC_50_ (nM)	
FK866	10.6	6,650	627
CHS-828	2.3	3,150	1,370
GNE-617	5.2	3,250	625
STF-118804	19.7	28,500	1,447

The cells were treated as described in the legend of Figure 3. The resistance index was determined as the EC_50_ values of HCT116R^FK866^ cells divided by the EC_50_ values of parental HCT116 cells. The EC_50_ values are average of triplicate determinations obtained at least 2 independent experiments. EC_50_, 50% effective concentration RI, resistance index: ^a^ *P <* 0.05, vs. HCT116 cells.

### Analysis of NAMPT mutations by whole-exon sequencing

To assess the relationship between cross-resistance to diverse NAMPT inhibitors and NAMPT mutations, we identified the point mutations in NAMPT in HCT116R^FK866^ cells by whole-exon next-generation sequencing. The results showed that two NAMPT mutations, H191R and K342R, were present in HCT116R^FK866^ cells (Table 1). In contrast, the parental HCT116 cells harbored only the K342R mutation. H191R is one of the six mutations previously reported in NAMPT inhibitor–resistant cells [20, 21]. These results suggest that cross-resistance to diverse NAMPT inhibitors is due to this mutation, and further that the H191R mutation may cause similar resistance to CHS-828 and STF-118804, but less resistance to GNE-617, in comparison with FK866. Furthermore, our data suggest that the degree of resistance by H191R mutation to various NAMPT inhibitors depends primarily on the head structures of those compounds, and less on the appended linker and tail groups (Figure 2).

### Biochemical features of the mutated NAMPT in HCT116R^FK866^

**Figure 4:**
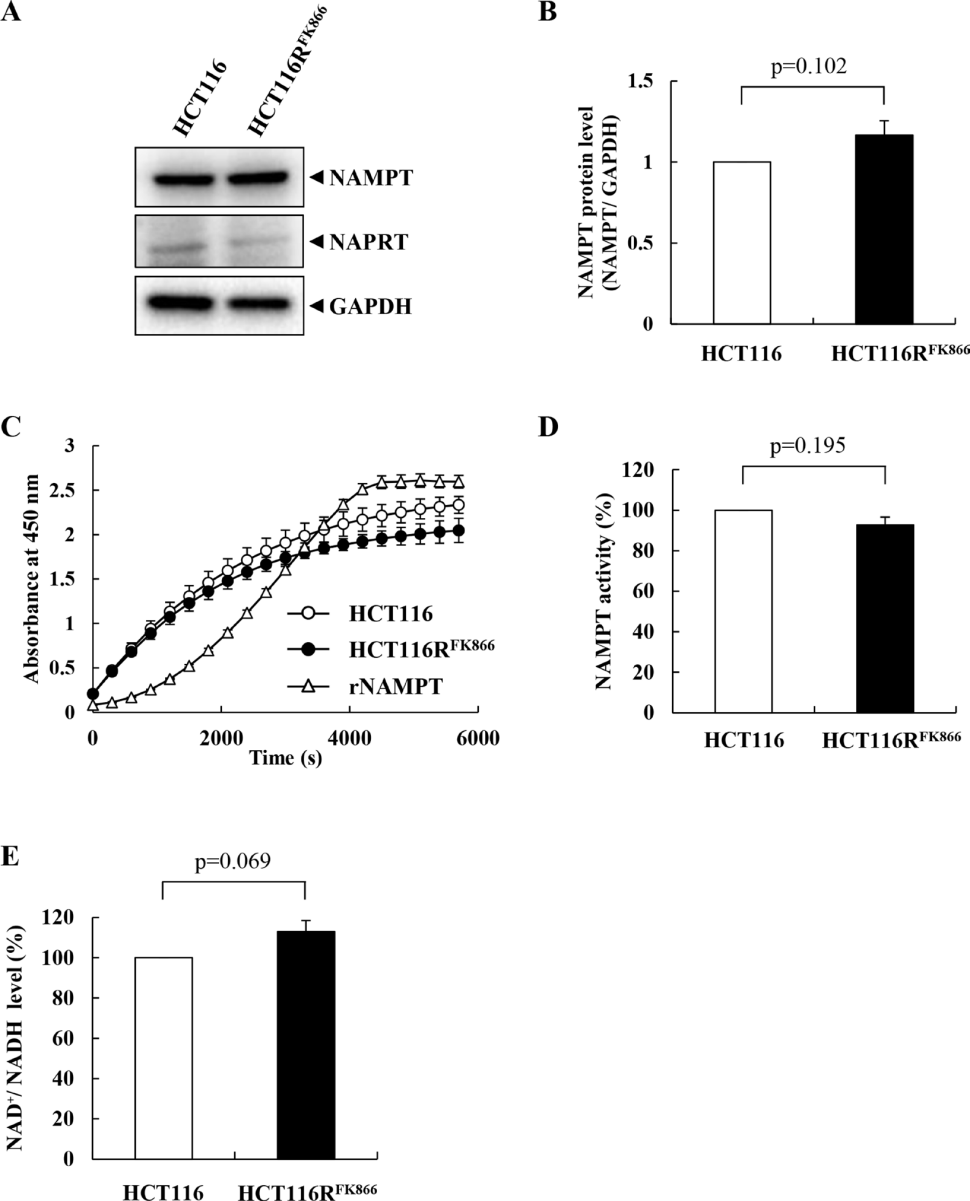
Characteristics of NAMPT in HCT116R^FK866^ and parental HCT116 cells (**A**, **B**) Protein levels of NAMPT, NAPRT, and GAPDH in HCT116R^FK866^ and parental HCT116 cells. (**C, D**) NAMPT enzyme activities of HCT116R^FK866^ and parental HCT116 cells were detected using a CycLex NAMPT colorimetric assay kit. (**E**) Intracellular NAD^+^/NADH levels of parental HCT116 and HCT116R^FK866^ cells. Results are averages of two independent experiments, with error bars showing ± SE of triplicates.

To elucidate the biochemical features of NAMPT in HCT116R^FK866^ and HCT116 cells, we monitored NAMPT expression levels by western blotting. As shown in Figure 4A and 4B, NAMPT protein levels were almost identical between the cell lines, as were the levels of nicotinic acid phosphoribosyltransferase (NAPRT1) (Figure 4A, middle) and glyceraldehyde-3-phosphate dehydrogenase (GAPDH), used as an internal control (Figure 4A, lower). We then measured NAMPT enzyme activities and intracellular NAD^+^ levels in HCT116R^FK866^ and HCT116 cells. As shown in Figure 4C and 4D, the NAMPT enzyme activities in cell lysates from HCT116R^FK866^ and HCT116 were almost the same. Similarly, the intracellular NAD^+^ levels were approximately equal in both cell lines (Figure 4E). These findings suggest that the NAMPT mutation in H191R primarily affects the NAMPT dimer conformation without impacting activity.

### Molecular mechanisms of cross-resistance to diverse NAMPT inhibitors

**Figure 5:**
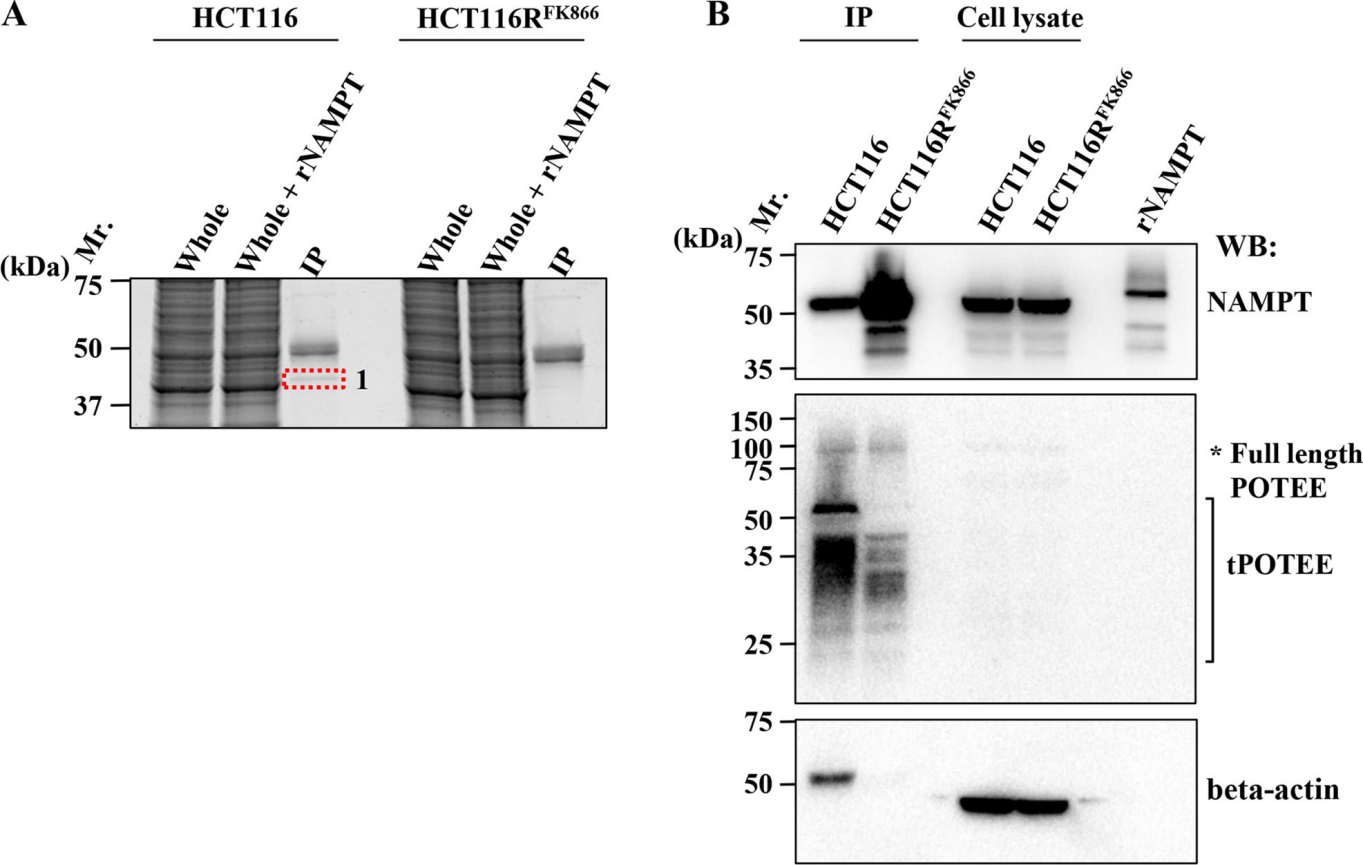
Identification of NAMPT-binding proteins (**A**) Immunoprecipitation (IP) experiments of HCT116R^FK866^ or parental HCT116 cell extracts were performed using mouse anti-NAMPT monoclonal antibody. IP samples were loaded on 12% SDS-PAGE and analyzed by Oriole fluorescent gel staining. Whole: whole-cell extracts; Whole + rNAMPT: whole-cell extracts plus recombinant NAMPT protein; IP: IP with mouse anti-NAMPT mAb. (**B**) Association of NAMPT and its binding proteins. After cell lysates were immunoprecipitated with mouse anti-NAMPT mAb, the precipitates were solubilized, and the supernatants were subjected to SDS-PAGE. Western blotting was carried out with rat anti-NAMPT (upper), rabbit anti- POTEE (middle), and mouse anti–beta actin (lower) antibodies, individually. Mr: protein size marker; rNAMPT, recombinant NAMPT protein; IP, IP with anti-mouse NAMPT mAb; Cell lysate: whole-cell lysate. Data are representative of at least three independent experiments.

To explore the molecular mechanisms by which the H191R mutation confers cross-resistance to various NAMPT inhibitors, we analyzed the NAMPT protein in FK866-resistant HCT116R^FK866^ and parental HCT116 cells by immunoprecipitation with anti-NAMPT mAb followed by proteomics analysis. The anti-NAMPT mAb precipitates were solubilized and analyzed by SDS-PAGE (Figure 5A). One protein band in the anti-NAMPT mAb immunoprecipitate (band 1), indicated by a red dotted line in Figure 5A, differed in intensity between HCT116R^FK866^ and HCT116 cells; accordingly, this band was cut out of the gel and analyzed by nano-LC-MS/MS. Two proteins, POTE ankyrin domain family member E (POTEE) and beta-actin, were identified in band 1 (Table 3). These results suggest that POTEE and beta-actin are specific NAMPT-binding partners in parental HCT116 cells.

**Table 3:** Identification of NAMPT binding proteins

Band no.	Identified protein	Accession no.^a^	Distinct peptides^b^	Score^c^	Sequence coverage	Theoretical MW
1	Actin, cytoplasmic 1	NP_001092.1	14	214	32	41,737
	POTE ankyrin domain family member E	NP_001077007.1	8	140	7	121,286

The protein analyzed (band 1) was excised from the silver stained gel and digested in gel trypsin methods. Peptides samples were analyzed by nano LC-MS/MS. ^a^ Accession number from NCBI protein database. ^b^ Number of matched peptides that in the an *in silico* the protein sequence. ^c^ Mascot score calculated for MS/MS results.

POTEE is a tumor-associated antigen that is expressed in a wide variety of human cancers, including breast, colon, lung, ovarian, pancreatic, and prostate tumors [22–24]. The level of POTEE is very low in normal tissues [23]. Actin, a ubiquitous protein in eukaryotes, is one of the major components of the cytoskeleton. Beta-actin, also known as a cytoplasmic actin, is predominantly expressed in nonmuscle cells, where it regulates cell structure and cell motility [25].

**Figure 6:**
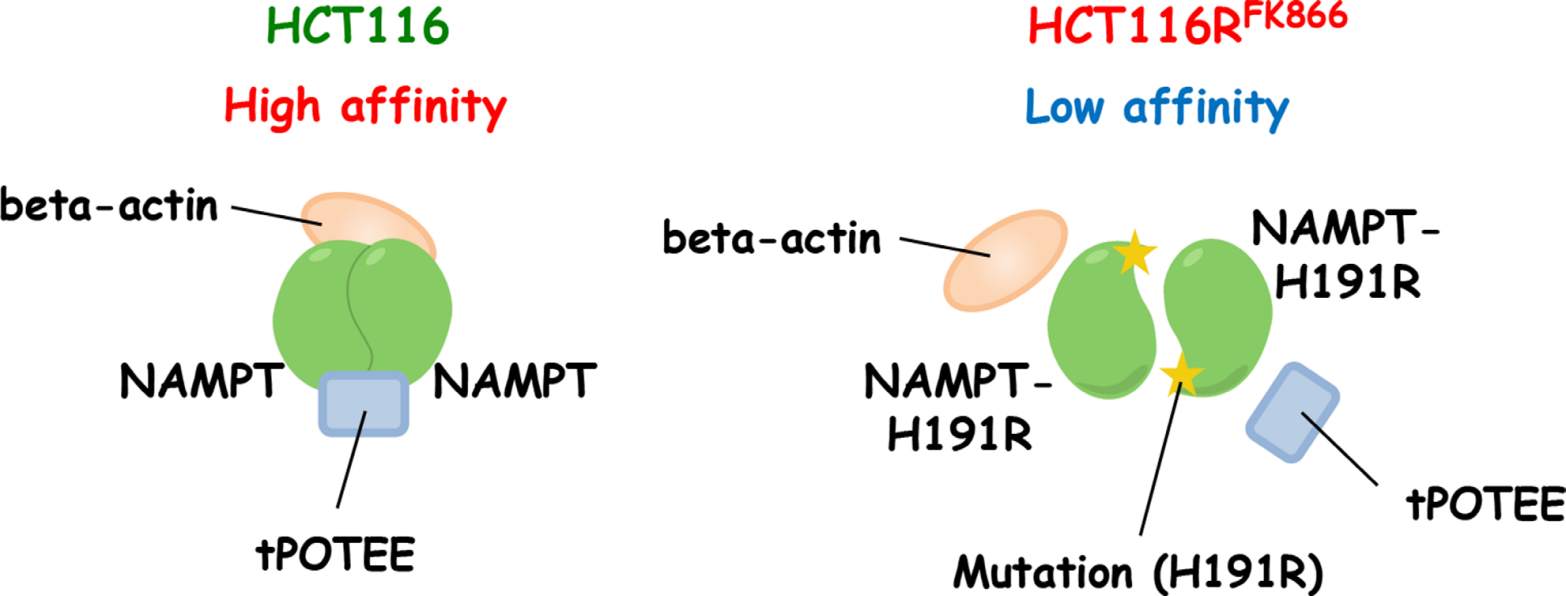
Predicted structure of NAMPT in HCT116R^FK866^ and parental HCT116 cells In HCT116 cells, NAMPT may be present in a homodimer in complex with tPOTEE and beta-actin, and have high affinity to NAMPT inhibitors. The H191R mutant in HCT116R^FK866^ cannot interact with the binding proteins, and consequently has lower affinity for diverse NAMPT inhibitors.

To validate the interaction between NAMPT and POTEE and/or beta-actin, we performed individual western blots using specific anti-NAMPT (Figure 5B, upper), anti-POTEE (Figure 5B, middle), or anti-beta actin antibody (mAbs) (Figure 5B, lower). Importantly, the amount of immunoprecipitated NAMPT protein was significantly higher in HCT116R^FK866^ cell extracts than in those from parental HCT116 cells (Figure 5B, upper). Full-length POTEE (around 121 kDa) was also observed (asterisk). Notably, a truncated POTEE (tPOTEE; indicated by a black line in Figure 5B, middle) of around 42 kDa was much more abundant in HCT116 than in HCT116R^FK866^ cells. Therefore, it is possible that POTEE undergoes post-transcriptional restricted proteolytic processing and post-translational modifications (e.g., ubiqutination or phosphorylation). In our experiments, the anti-rabbit HRP-conjugated secondary antibodies were not cross-reacted with immunoglobulin heavy and light chain of the immunoprecipitated mouse anti-NAMPT monoclonal antibody. Thus, we assume that the smear-like bands immunoblotted by rabbit anti-POTEE antibodies were not due to artifact such as immunoglobulins use for the immunoprecipitation (Figure 5B, middle). In addition, the level of beta-actin in the immunoprecipitants was significantly higher in HCT116 than in HCT116R^FK866^ cells (Figure 5B, lower). However, the beta-actin protein levels in whole-cell extracts did not differ significantly between the two cell lines (Figure 5B, lower). We consider that the different position of beta-actin band in IP or cell lysate lane was caused by differences the proteins composition in IP or whole cell lysate (Figure 5B lower). The H191R mutation in NAMPT is located in the binding site for NAM and NAMPT inhibitors (*e.g.*, FK866, CHS-828, and TP201565) and in the NAMPT dimer interface [8, 20, 21]. These observations suggest that the NAMPT H191R variant in HCT116R^FK866^ cells fails to achieve complete dimer formation or interact with its binding partner(s), tPOTEE, and/or beta-actin, resulting in lower affinity for diverse NAMPT inhibitors (Figure 6). Interestingly, these data shows that the differences of NAMPT protein complex, interactions with or without binding partners, tPOTEE and beta-actin, between parental HCT116 and FK866-resistant HCT116R^FK866^ cells do not affect the NAMPT activities.

## MATERIALS AND METHODS

### Reagents

NAMPT inhibitors FK866, CHS-828, GNE-617, and STF-118804 were obtained from Focus Biomolecules, Cayman Chemical, MedChem Express, and Toronto Research Chemicals, respectively. FK866 hydrochloride was stored as a 10 mM stock in ultra-pure water at -20°C. CHS-828, GNE-617, and STF-118804 were stored as 10 mM stocks in dimethyl sulfoxide (DMSO) at -20°C.

### Cell culture

The human colon cancer cell line HCT116 was obtained from the American Type Culture Collection. Parental and resistant HCT116 cell lines were cultured in D-MEM containing 10% heat inactivated fetal bovine serum, 100 units/mL penicillin, and 100 µg/mL streptomycin in a 37°C incubator under an atmosphere containing 5% CO_2_ at 100% relative humidity.

### Generation of an FK866-resistant HCT116 cell line (HCT116R^FK866^)

FK866-resistant HCT116 cells were obtained by continuously exposing cells to increasing concentrations of FK866, between 3, 6, 30, 60, 300, and 600 nM, over a 6 weeks period. A derivative of HCT116 was isolated and named HCT116R^FK866^. The HCT116R^FK866^ cells were maintained in culture in the presence of 600 nM FK866. No mutagenic agents were used to establish the FK866-resistant HCT116 cells.

### Cell viability assay

Cell viability was determined using the WST-8 cell proliferation assay (Takara Bio, Otsu, Japan). Briefly, cells were passed into 96-well plates (1,000 cells per well) in triplicate, and then treated with various concentrations of drugs or ultra-pure water (as a negative control). Following incubation for 72 hr, 20 μL of WST-8 reagent was added to each well, and the plate was placed in a 5% CO_2_ incubator at 37°C for an additional 1 hr. Optical density was measured at 490 nm on a Tecan microplate reader. The EC_50_ value was defined as the concentration of drug producing 50% inhibition of cell proliferation. The RI was defined as the ratio of EC_50_ values between the resistant and parental cell lines. Experiments were repeated at least three times.

### Colony formation assay

Colony formation assay was performed as described previously [26, 27]. HCT116 and HCT116R^FK866^ cells were dissociated with Accutase, suspended in medium, inoculated into six-well plates (200 cells per well) in triplicate, and then incubated overnight. The cells were treated with various concentrations of drugs or with solvent (DMSO or ultra-pure water) as a negative control. After incubation for 10 days, cells were fixed with 4% formaldehyde solution and stained with 0.1% (w/v) crystal violet, and then the number of colonies in each well was counted.

### Analysis of NAMPT activity

NAMPT activity was assayed using whole-cell lysates prepared by incubating cells in lysis buffer (2% CHAPS in PBS containing protease-inhibitor cocktail [Roche]) on ice for 30 min. Cell lysates were sonicated on a Branson Sonifier-250, then centrifuged at 15,000 ×g for 15 min at 4°C. The supernatants containing the solubilized protein fractions were subjected to enzymatic assays. NAMPT activity was measured using a coupled-enzyme reaction system (CycLex NAMPT colorimetric assay kit) in 96-well plate format using the one-step method.

### Measurement of intracellular NAD^+^/NADH levels

Parental HCT116 and HCT116R^FK866^ cells were dissociated with Accutase and suspended in PBS, and the cell suspensions were subjected to NAD^+^/NADH assays using the NAD/NADH-Glo Assay kit (Promega). The resulting luminescent signals were measured on a Tecan microplate reader.

### Whole-exon sequencing analysis

DNA extraction was performed as described previously [28]. Genomic DNA was extracted from cells (5 × 10^6^ cells per sample) using the DNeasy Tissue Kit (QIAGEN). Whole-exon sequencing of parental HCT116 and HCT116R^FK866^ cells was performed by the APRO Life Science Institute, Inc. (Tokushima, Japan) and Macrogen Global Headquarters (Seoul, Korea).

### Immunoprecipitation

Immunoprecipitation was performed as described previously [29]. HCT116 and HCT116R^FK866^ cells were gently lysed with lysis RIPA buffer (50 mM Tris-HCl, pH 8.0, 150 mM sodium chloride, 1.0% NP-40, 0.5% sodium deoxycholate, and 0.1% SDS; product number: R0278; Sigma-Aldrich, St. Louis, MO, USA) with protease and phosphatase inhibitors (Roche), sonicated on a Branson Sonifier-250, and then centrifuged at 15, 000 ×g at 4°C for 15 min. The supernatant, collected as cell lysate (equivalent to ∼3 × 10^6^ cells), was treated with 30 μL of protein A-sepharose beads (Invitrogen) to remove nonspecifically binding proteins, and then incubated with 2 µg of mouse anti-NAMPT mAb (CycLex) at 4°C for 4 hr. The lysate was then incubated with an additional 30 μL of protein A agarose beads at 4°C for 12 hr. The immunoprecipitated complexes were washed three times with RIPA buffer. NAMPT–protein complexes were eluted from beads by heating at 100°C for 5 min in 2× Laemmli sample buffer (62.5 mM Tris-HCl, pH 6.8, 25% glycerol, 2% SDS, and 0.01% bromophenol blue; #161–0737, Bio-Rad, Hercules, CA, USA) supplemented with 10% beta- mercaptoethanol, and then subjected to western blot analysis.

### Protein identification

Protein identification was subjected to nano-LC/MS/MS analysis according to a standard protocol [29, 30].

### Western blotting

Western blot analysis was performed as described previously [30, 31]. The following antibodies were used: rat anti-NAMPT clone 14A.5 (1:1,000, Millipore), rabbit anti-GAPDH (1:20,000, Trevigen), mouse anti-NAPRT(B-8) (1:1,000, Santa Cruz Biotechnology), rabbit anti-POTEE antibody (1:200, Abcam), mouse monoclonal anti-beta actin clone AC-15 (1:20,000, Sigma-aldrich), horseradish peroxidase-linked anti-rat IgG (1:20,000, GE Healthcare), horseradish peroxidase-linked anti-rabbit IgG (1:20,000, GE Healthcare), and horseradish peroxidase-linked whole antibody anti-mouse IgG (1:20,000, GE Healthcare).

### Statistical analysis

The data are presented as the means ± standard deviation. Significance of differences among groups was evaluated using Student’s *t*-test; *P* < 0.05 was considered to indicate a statistically significant difference.

## CONCLUSIONS

In this study, we generated a cancer cell line that is cross-resistant to diverse NAMPT inhibitors, identifying the mutation responsible for resistance, and elucidated the molecular mechanisms of action. Our work revealed that mutation of H191R in the NAMPT molecule is responsible for cross-resistance to diverse NAMPT inhibitors. Interestingly, in immunoprecipitation analysis with anti-NAMPT mAb, the amount of immunoprecipitated NAMPT protein was significantly higher in HCT116R^FK866^ cell extracts. We also identified two novel NAMPT-binding proteins, POTEE and beta-actin, in parental HCT116 but not in HCT116R^FK866^ cells. Our data suggest that the NAMPT H191R mutant in HCT116R^FK866^ fails to achieve complete dimer formation due to diminished interactions with its binding partners, POTEE and/or beta-actin, and consequently has lower binding affinity to NAMPT inhibitors. Analysis of the modes of action of these partners should facilitate development of new classes of NAMPT inhibitors and novel anticancer therapeutic strategies.

## Abbreviations

NA: nicotinic acid
NAM: nicotinamide
NAMPT: nicotinamide phosphoribosyltransferase
NAPRT1: nicotinic acid phosphoribosyltransferase
NMN: nicotinamide mononucleotide
POTEE: POTE ankyrin domain family member E
PRPP: phosphoribosyl pyrophosphate
